# Sensitivity of a national coronial database for monitoring unnatural deaths among ex-prisoners in Australia

**DOI:** 10.1186/1756-0500-4-450

**Published:** 2011-10-27

**Authors:** Jessica Y Andrews, Simon Forsyth, Jessica Wade, Stuart A Kinner

**Affiliations:** 1 Centre for Population Health, Burnet Institute, 85 Commercial Road, Melbourne, VIC Australia; 2 School of Population Health, University of Queensland, Herston Road, Herston, QLD Australia; 3 School of Public Health and Preventative Medicine, Monash University, 85 Commercial Road, Melbourne, VIC Australia

## Abstract

**Background:**

The period immediately after release from custody is a time of marked vulnerability and increased risk of death for ex-prisoners. Despite this, there is currently no routine, national system for monitoring ex-prisoner mortality in Australia. This study subsequently aimed to evaluate the sensitivity of Australia's National Coroners Information System (NCIS) for identifying reportable deaths among prisoners and ex-prisoners.

**Findings:**

Prisoner and ex-prisoner deaths identified through an independent search of the NCIS were compared with 'gold standard' records of prisoner and ex-prisoner deaths, generated from a national monitoring system and a state-based record linkage study, respectively. Of 294 known deaths in custody from 2001-2007, an independent search of the NCIS identified 229, giving a sensitivity of 77.9% (72.8%-82.3%). Of 677 known deaths among ex-prisoners from 2001-2007, an independent search of the NCIS identified 37, giving a sensitivity of 5.5% (4.0-7.4%). Ex-prisoner deaths that were detected were disproportionately drug-related, occurring within the first four weeks post-release, among younger prisoners and among those with more than two prior prison admissions.

**Conclusions:**

Although a search of the NCIS detected the majority of reportable deaths among prisoners, it was only able to detect a small minority of reportable deaths among ex-prisoners. This suggests that the NCIS is not effective for monitoring mortality among ex-prisoners in Australia. Given the elevated rates of mortality among ex-prisoners in Australia and elsewhere, there remains an urgent need to establish a process for routine monitoring of ex-prisoner mortality, preferably through record linkage.

## Background

The period immediately after release from custody is a time of marked vulnerability and increased risk of death for ex-prisoners. The risk of death is greatest in the weeks immediately following release, and the majority of deaths during this time are attributed to preventable, reportable causes such as drug overdose and suicide [1-5].

Deaths in custody are less common [6], however in Australia these deaths have been monitored on an annual basis since 1992 through the National Deaths in Custody Program (NDICP) [7]. More recently, the health of prisoners has been the subject of annual monitoring through a national minimum dataset (NMDS) [8]. The NMDS includes one 'aspirational indicator' of deaths among ex-prisoners, however national data for this indicator are not currently available [8].

Studies investigating ex-prisoner mortality have typically used record linkage methods, matching correctional data with a state-based or national death register, such as the Australian National Death Index (NDI) [3,5]. Although these studies have provided valuable insights into the incidence and risk factors for mortality in ex-prisoners, routine record linkage of this sort it is not currently feasible at a national level in Australia [9,10].

One alternative source of data for monitoring mortality among ex-prisoners is coronial records. In Australia, the National Coroners Information System (NCIS) contains information on all deaths reported to a coroner since 2001 [11]. While what constitutes a reportable death varies by Australian jurisdiction, a death is generally reported to a coroner where: (1) the persons dies unexpectedly and the cause of death is unknown; (2) the person died in a violent or unnatural manner; (3) the person died during or as a result of anaesthetic; (4) the person was 'held in care' or in custody immediately before they died; (5) a doctor has been unable to sign a death certificate giving the cause of death; or (6) the identity of the person who died is unknown [11]. Previous studies have utilised coronial records to examine the circumstances surrounding deaths among ex-prisoners [12,13], but none have considered the utility of the NCIS for monitoring mortality among ex-prisoners in Australia. Given the preponderance of reportable deaths among ex-prisoners, many deaths in this group are the subject of a coronial inquiry and should thus be recorded in the NCIS.

The aims of this study were to establish what proportion of reportable deaths among ex-prisoners (and for comparison purposes, prisoners) in Australia could be detected through a comprehensive search of the NCIS, and to assess the representativeness of deaths detected through such a search.

## Methods

### NCIS case identification

To identify reportable deaths among prisoners and ex-prisoners, the NCIS was interrogated using the 'keyword search' function, with the terms 'custod*', 'correction*', 'incarcerat*', 'prison*', 'inmate', 'gaol', 'jail', 'probation', 'parole', 'remand' and 'sentence'. Persons in custody were further identified using a text search for 'prison' and 'inmate' in the occupation text field. Searches were restricted to deaths from 2001-2007 based on the availability of death registration numbers, and 'open' cases were excluded due to restriction of case-related information. The NCIS keyword search is current as of June 2009.

A deceased prisoner was defined as a person held in an adult prison (sentenced or remand) or a secure/prison hospital, or serving home, weekend or periodic detention at the time of death. A deceased ex-prisoner was defined as any individual who had died in the community but had previously been a prisoner according to the above criteria. Deaths in police custody and cases that referred to a criminal history or previous criminal charges, but did not clearly indicate a previous incarceration episode, were excluded.

NCIS case information was extracted by two research assistants from 2009-2010. To ensure coding consistency, 1 in 10 records were double-coded throughout the data collection process. Any discrepancies identified in coding were re-coded according to a final consensus.

### Deaths in custody

Data extracted from the NDICP from 2001-2007 were compared with prisoner deaths identified through the NCIS search. To ensure that prisoner deaths identified through the NCIS search were comparable with NDICP data, juvenile deaths were included, while deaths among persons on home, weekend or periodic detention were excluded.

### Deaths among ex-prisoners

Deaths among persons previously incarcerated in the state of Queensland, Australia were identified through probabilistic linkage of Queensland correctional records with the Australian NDI. Linkage was performed by the Data Linkage Unit at the Australian Institute of Health and Welfare (AIHW) using probabilistic matching based on name(s), date(s) of birth, sex, last contact state and date last known alive. Deaths were identified for any prisoner released from a Queensland correctional facility from 1994-2007, resulting in 2,386 ex-prisoner deaths nationally.

For the purposes of this study, these records were then linked with NCIS records using deterministic matching based on a combination of death registration number, state of death registry, date of birth, and date of death being equal to or prior to the year of coronial exam. This resulted in the identification of a subset of 677 ex-prisoner deaths reported to an Australian coroner from 2001-2007. Reportable ex-prisoner deaths identified through this linkage process were then compared with reportable ex-prisoner deaths identified through the NCIS search to establish the proportion of reportable deaths among ex-prisoners in Australia detected through a comprehensive search of the NCIS.

### Ethics

Ethical approval for the research was obtained from the Victorian Department of Justice Research Ethics Committee, the University of Queensland's Behavioural and Social Sciences Ethical Committee, and the State Coroner Western Australia Coronial Ethics Committee.

## Results

The NCIS search yielded 409 deaths among ex-prisoners and 247 deaths in custody from 2001-2007.

### Deaths in custody

The NDICP reported 294 deaths in prison from 2001-2007, while the NCIS search identified 229 prisoner deaths over the same period, giving a sensitivity of 77.9% (CI: 72.8%-82.3%). Excluding data from 2007, in which there was a higher proportion of open cases, increased the sensitivity to 85.6% (80.6%-89.4%).

### Deaths among ex-prisoners

Reportable ex-prisoner deaths identified through record linkage (N = 677) were compared with those identified independently through the NCIS search (n = 409), resulting in a match of 37 cases and a sensitivity of 5.5% (CI: 4.0%-7.4%). Of these, the vast majority involved non-indigenous males and deaths were relatively evenly distributed over time. In a multivariate logistic regression model, the odds of being identified as an ex-prisoner death through the NCIS search were significantly greater for those who died within 28 days of release (OR = 7.39, 95%CI = 3.03, 18.04), for those with more than two prior prison admissions (OR = 2.43, 95%CI = 1.18, 5.02), for those less than 30 years old at the time of death (OR = 3.44, 95%CI = 1.65, 7.17) and for those whose death was reported as drug-related (OR = 3.23, 95%CI = 1.56, 6.71) (Table 1).

**Table 1:** Factors associated with an ex-prisoner case being identified by NCIS search

	Identified in NCIS search		Unadjusted OR (95% CI)	Adjusted OR (95% CI)
			
	No (n = 640) %	Yes (n = 37) %		
Age at death < 30 years	23.0	48.7	3.35 (1.71, 6.56)	3.44 (1.65, 7.17)
Non-Indigenous	81.1	83.8	1.21 (0.49, 2.96)	
> 2 prison admissions	29.7	51.4	2.50 (1.28, 4.87)	2.43 (1.18, 5.02)
Death within 28 days after release	4.1	27.0	8.75 (3.83, 19.96)	7.39 (3.03, 18.04)
Primary cause of death is drug related	18.4	48.7	4.19 (2.13, 8.23)	3.23 (1.56, 6.71)

## Discussion

Although a search of the NCIS detected the vast majority of reportable deaths among prisoners, we were able to detect only a small minority of reportable deaths among ex-prisoners. Those deaths that were detected were disproportionately drug-related, occurring within the first four weeks post-release, among younger prisoners and among those with more than two prior prison admissions.

Although a number of previous studies have used coronial records to study deaths among ex-prisoners [12,13], our findings are not unexpected, particularly given the nature of the coronial process. The primary objective of a coroner is to determine relevant circumstances surrounding a reportable death [11]; it is not to facilitate identification of ex-prisoner deaths. The NCIS is therefore likely to underestimate reportable ex-prisoner deaths, particularly those that occur as more time elapses after release from custody.

Despite this, there was a bias towards reporting those deaths that were drug-related, that occurred within four weeks of release, among younger prisoners and among those with more than two prior prison admissions. The increased detection of these deaths, based on reference to ex-prisoner status in their coronial records, may reflect a growing understanding among those involved in the coronial process of the risk of drug-related death in the weeks immediately following release from custody, particularly for those with multiple prior imprisonments [14,15].

This study had two main limitations. Firstly, given the de-identified nature of NDICP reports, our comparison of prisoner deaths detected by the NDICP and NCIS search was based on the assumption that these were the same individuals. Although it is highly unlikely that the NCIS search would have detected prisoner deaths not recorded in the NDICP, we were unable to verify this. Secondly, issues of uncertainty and subjectivity with qualitative 'free-text' data meant that we adopted a conservative decision rule to minimise the rate of ex-prisoner inclusions assessed as false positives. For example, cases were excluded if any reference(s) to a previous incarceration history were uncertain or could be interpreted subjectively i.e. 'an extensive criminal history'. This resulted in the exclusion of 52 ex-prisoner deaths. However, even after including these additional cases, our NCIS search identified only 13.1% of known ex-prisoner records in the NCIS.

## Conclusions

Given the markedly elevated risk of death after release from custody, there is an urgent need to develop a system for regular monitoring of mortality among ex-prisoners in Australia. One efficient and reliable approach would be through routine record linkage, however this is not currently possible in any Australian jurisdiction. An alternative approach involves routine interrogation of coronial records through the NCIS, however the findings of this study indicate that the NCIS is able to identify only a small and biased subset of ex-prisoner deaths, rendering it unsuitable for monitoring purposes. Nonetheless, the NCIS remains a rich and largely untapped source of information for exploring the causes and contexts in which deaths among ex-prisoners occur.

## Abbreviations

AIHW: Australian Institute of Health and Welfare; NCIS: National Coroners Information System; NDI: National Death Index; NDICP: National Deaths in Custody Program; NMDS: National Minimum Dataset

## Competing interests

The authors declare that they have no competing interests.

## Authors' contributions

JA participated in data collection and drafted the manuscript. SF performed the statistical analysis. JW participated in data collection and helped to draft the manuscript. SK conceived the study, and participated in its design and coordination and helped to draft the manuscript. All authors read and approved the final manuscript.
